# Editorial: “Small molecules targeting transmembrane receptors and ion channels in drug discovery”

**DOI:** 10.3389/fmolb.2023.1183713

**Published:** 2023-05-02

**Authors:** Rosa Maria Vitale, Fabio Arturo Iannotti, Tullio Florio

**Affiliations:** ^1^ Institute of Biomolecular Chemistry-National Research Council (ICB-CNR) of Italy, Pozzuoli, Italy; ^2^ Section of Pharmacology, Department of Internal Medicine, University of Genova, Genova, Italy; ^3^ IRCCS Policlinico San Martino, Genova, Italy

**Keywords:** drug discovery, small molecules, GPCRs, ion channels, in silico studies, receptor conformation

This Research Topic (RT) is a collection of contributions focused on either the discovery of bioactive molecules targeting specific ion channels or the analysis of structural and dynamic features of relevant molecular targets, potentially useful to guide the screening and the rational design of novel leads.

In the broad scenario of druggable molecules, natural compounds represent a continuous source of inspiration for medicinal chemists due to the high diversity of their chemical scaffolds. Natural compounds also led to the discovery and characterization of relevant molecular targets, as, for example, in the case of phytocannabinoids with cannabinoid receptors, or capsaicin and menthol with TRPV1 and TRPM8 channel receptors, respectively.

Allicin, the major bioactive compound of garlic, has been extensively studied for its bactericidal activity against pulmonary pathogens and for its lung-protective effects (Reiter et al., 2017; Shen et al., 2019). In this RT, Qui et al. have found that allicin stimulates Cl^−^ and fluid secretion across airway epithelium via activation of cystic fibrosis transmembrane conductance regulator (CFTR) providing novel insights into the physiological function of allicin in the respiratory system.

From a general point of view, the identification and/or the lead-to-hit optimization of novel bioactive molecules is a complex and multidisciplinary process which takes advantage from structural insights arising from an ever increasing number of available three-dimensional structures of their molecular targets. Transmembrane proteins such as G-protein coupled receptors (GPCRs), transient receptor potential (TRP) channels and voltage- or ligand-gated ion channels, cover a relevant portion of druggable targets due to their key role in a wide range of pathological processes ranging from pain, inflammation, cancer, metabolic to neurological and neuropsychiatric disorders. Transmembrane receptors and ligand-gated ion channels are subject to modulation at multiple sites by both orthosteric and allosteric ligands. The occurrence of multiple binding sites and their mutual coupling add further complexity to their mechanism of action which usually requires large conformational transitions to switch from inactive to active states. Such structural rearrangements involve single helices as in the case of GPCRs up to whole monomers for ligand-gated ion channels.

A strategy to stabilize GPCRs in distinct conformations to facilitate the *in vitro* screening, or decipher their complex pharmacology and signaling, is based on camelid-derived immunoglobulin single variable domains (VHHs or ConfoBodies). VHHs stabilize receptor conformations by interacting with their cytosolic region either directly or indirectly. The applications of this approach have been reviewed in this) RT by Laeremans et al.

Among the class A of GPCRs, μ-opioid represent the primary targets for opioid drugs being involved in the control of pain and reward properties (Ugur, Derouiche, and Massotte, 2018). Zádor et al. , have contributed to this RT by describing how *in silico* studies, either alone or in combination with experimental techniques such as NMR or mutagenesis, greatly contributed to unveiling the molecular determinants responsible for the binding and activation of μ-opioid receptors. They have also illustrated the fundamental role of advanced molecular dynamics approaches and NMR to the characterization of large-scale conformational transitions associated with receptor activation.

A relevant Research Topic in drug discovery, frequently preventing the development of highly selective ligands, is the high degree of conservation of the binding sites for proteins belonging to the same family or sub-family. Filip Koniuszewski et al. have contributed to this RT by performing a comparative structural study on a selection of allosteric binding sites - for which *in vitro* screening is lacking - of cys-loop receptors. This is a family of pentameric ligand-activated ion channels which comprises acetylcholine receptors (nAChRs), 5-hydroxytryptamine type 3 receptors (5-HT3Rs), zinc activated channels (ZAC), γ-aminobutyric acid type A receptors (GABAARs) and glycine receptors (GlyRs). The study combines different computational methods with the goal to improve the efficiency of *in silico* screening.

Another therapeutically relevant class of ion channels is represented by K+ ion channels (Kvs). Targeting Kv1.5 is considered an effective strategy for the treatment of re-entrant based atrial fibrillation, being highly expressed in human cardiac atria, but scarcely in ventricles, as well as for the treatment of tumors overexpressing this subtype of ion-channel. The last contribution of this RT is from Dong et al. These authors, through a multidisciplinary approach based on molecular docking, mutagenesis and whole-cell patch-clamp techniques have shown that HMQ1611, a taspine derivative with anticancer effects *in vitro* and *in vivo* breast cancer models (Zhan et al., 2012), acts as a human Kv1.5 channel blocker by reversibly inhibiting the underlying outwardly K+ currents in a concentration-dependent manner.
